# Community acquired paediatric pneumonia; experience from a pneumococcal vaccine- naive population

**DOI:** 10.1186/s41479-020-00071-6

**Published:** 2020-08-25

**Authors:** Sanath Thushara Kudagammana, Ruchira Ruwanthika Karunaratne, Thilini Surenika Munasinghe, Haputhanthirige Donna Wasana Samankumari Kudagammana

**Affiliations:** 1 Department of Paediatrics, Faculty of Medicine, University of Peradeniya, Peradeniya, 20400 Sri Lanka; 2 Department of Medical Laboratory Sciences, Faculty of Allied Health sciences, University of Peradeniya, Peradeniya, Sri Lanka

**Keywords:** Community acquired pneumonia, Paediatric pneumonia, Pneumococcal pneumonia

## Abstract

**Background:**

Childhood pneumonia continues to be a disease that causes severe morbidity and mortality among children mainly in South-East Asia and Africa though it is not so in the developed world. Pneumonia accounts for 16% of all deaths of children under 5 years old in the world, killing nearly one million children in 2015. In Sri Lanka, there were 21,000 reported cases of pneumonia in 2006, 40% were in the age group of less than 4 years.

**Methods:**

This was a retrospective study done on the children aged 1 month to 14 years who were admitted to the Professorial Paediatric unit of Teaching Hospital, Peradeniya between 1st of March 2016 and 30th of July 2017 fulfilling diagnostic criteria for community-acquired pneumonia. Data including diagnosis, clinical details, management details and other relevant data were collected from patient records by using a data collection sheet.

**Results:**

In this study, 48% of 127 patients admitted with community-acquired pneumonia had bronchopneumonia. About 2/3 of the patients neededa secondline of intravenous antibiotics while 51/ 127 needed care in the high dependency unit with supplemental oxygen. No mortality was observed in the group.

**Conclusions:**

Community- acquired paediatric pneumonia has a significant associated morbidity but not mortality in the studied population. The need for the second-line treatment with intravenous antibiotics in a significant proportion of patients may indicate a high degree of antibiotic resistance. Introduction of national antibiotic policy will help the cause.

## Background

Childhood pneumonia continues to be a disease that causes severe morbidity and mortality among children mainly in South-East Asia and Africa though it is not so in the developed world. Pneumonia accounts for 16% of all deaths of children under 5 years old in the world, killing nearly one million children in 2015. In Sri Lanka, there were 21,000 reported cases of pneumonia in 2006, 40% were in the age group of less than 4 years [1].

Pneumonia is defined as inflammation of the lung parenchyma due to infection leading to consolidation of pulmonary tissue. When pneumonia is acquired in the community by a previously well person it is defined as community-acquired pneumonia [2].

Pneumonia can also be categorised as bronchopneumonia and lobar pneumonia according to the patho-physiological basis [3] and viral, bacterial, fungal or tuberculous pneumonia on an aetiological basis [4]. Atypical pneumonia is caused by Mycoplasma and Chlamydia compared to typical organisms causing pneumonia and is commonly seen in pre-school and school-going children [5]. Pneumonia remains the single most common cause of death in childhood in the developing world [6].

Sri Lanka is a low middle-income country, having significantly better health indices compared to many economically comparable countries. Most of the Sri Lankan population has easy access to a health care delivery institution manned by trained medical officers [7]. All the citizens of the country enjoy free access to complete health care in government healthcare institutions. In parallel to this, there is a significant number of academically qualified private medical practitioners and many private hospitals, mainly in bigger cities [8].

The country has a well-developed hospital system. Each of the nine administrative provinces has a Provincial General Hospital served by consultants of major specialities and many subspecialties [8, 9]. There is a network of hospitals where these hospitals get referrals from District General Hospitals, Base hospitals and smaller District Hospitals. The former two are manned by a consultant paediatrician. The bigger hospitals have intensive care facilities and some of them having dedicated intensive care facilities for children [10]. All of these hospitals have a linking ambulance service where patients can be transferred from one institution to another when required. Patients have access to any hospital of their choice for their health care needs.

The country also boasts of a very good preventive health care system with immunisation coverage above 90% [11, 12]. This system is applauded as a system of similar quality to a developed country. The national immunisation schedule includes vaccination against *Haemophillus influenzae* B but not against pneumococcal disease.

Guidelines to manage community-acquired pneumonia vary in different countries according to the available resources and the likelihood of resistant organisms in the affected population. WHO has formulated guidelines to diagnose and manage pneumonia in resource-poor settings. These guidelines recommend considering those children presenting with tachypnoea as having pneumonia and to treat them with antibiotics at home. Those who had chest retractions are recommended to be admitted to a higher health authority for intravenous antibiotics [13].

British Thoracic (BTS) guidelines recommend those with a mild form of pneumonia to be managed at home with oral antibiotics. Those who have severe disease, those who deteriorate, are complicated or those who do not tolerate oral medication need to be admitted for in-house intravenous antibiotics [14].

The same guidelines recommend those children needing admission to the hospital for intravenous antibiotics to have cefuroxime, cefotaxime or co-amoxiclav as best-guess antibiotics. Those who are very ill and those with poor response are recommended to receive a macrolide added to the antibiotic regime. They need to be followed up for complications and recovery [14].

Acute phase reactants are not recommended routinely as investigations. So is a chest x-ray. As viral pneumonia are not easily differentiated from bacterial, all cases are recommended to be treated as bacterial pneumonia [15, 16].

Our study was conducted in the Teaching hospital Peradeniya which is a premier Teaching hospital of the country getting direct admissions through the busy outpatient department of the hospital and those referred from other regional hospitals. The hospital has an 80-bedded acute paediatric ward and a 6-bedded high dependency unit. Those needing intensive care are transferred to the ICU in the hospital or elsewhere depending on the facilities available. The unit has its Admission Guidelines developed on the Infectious Diseases Society of America (IDSA) Paediatric Pneumonia Guidelines and Management Guidelines developed according to British Thoracic Society (BTS) Guidelines. Guidelines to transfer to ICU care are made on IDSA Paediatric Guidelines.

There is a paucity of data regarding childhood pneumonia in the developing world. This study is an effort to fill up the dearth of information in some aspects of this condition, in this pneumococcal vaccine-naive population in a country with high health indices and a well-developed health care system accessible to most of the population.

## Methods

The aim of the study was to describe the outcome of those children with acute community-acquired pneumonia getting admitted to the Teaching Hospital Peradeniya, Sri Lanka. This was a retrospective study done on the children who were admitted to the Professorial Paediatric Unit of Teaching Hospital, Peradeniya between1stof March 2016 and30^th^ of July 2017, fulfilling diagnostic criteria for community-acquired pneumonia [14] and Admission Guidelines of the unit. All those children who have physician-diagnosed community- acquired pneumonia fulfilling BTS guidelines were included in the study. Data including diagnosis, drug history and other relevant data were collected from patient records by using a data collection sheet. The chest x-ray films and baseline and follow-up blood count done on them were also perused. Children from the age of completed 1 month to 14 years were included into the study.

## Results

One hundred and twenty-seven (127) children fulfilling the inclusion criteria were included in this study. The total number of acute admissions during the study period was 4447. 58% of the study population consisted of girls and 42% of boys. The commonest age group affected was 1–5 years age group consisting of 69 patients. Forty two patients were in the age group of 1 month to 1 year and 15 patients were above 5 years.

A Consultant Paediatrician has made the diagnosis in all the cases. In 70% of them, the clinical diagnosis was supplemented by laboratory evidence (white cell count and C reactive protein) and in 30% supplemented by radiological and laboratory-based evidence.

Majority of the patients (102/127, 80%) had got admitted during the first 10 days of the illness and 55/127(43%) of the children had been admitted to the hospital within the first 1–4 days.25/127 (19.8%) had got admitted after 10 days of the illness.

**Fig. 1:**
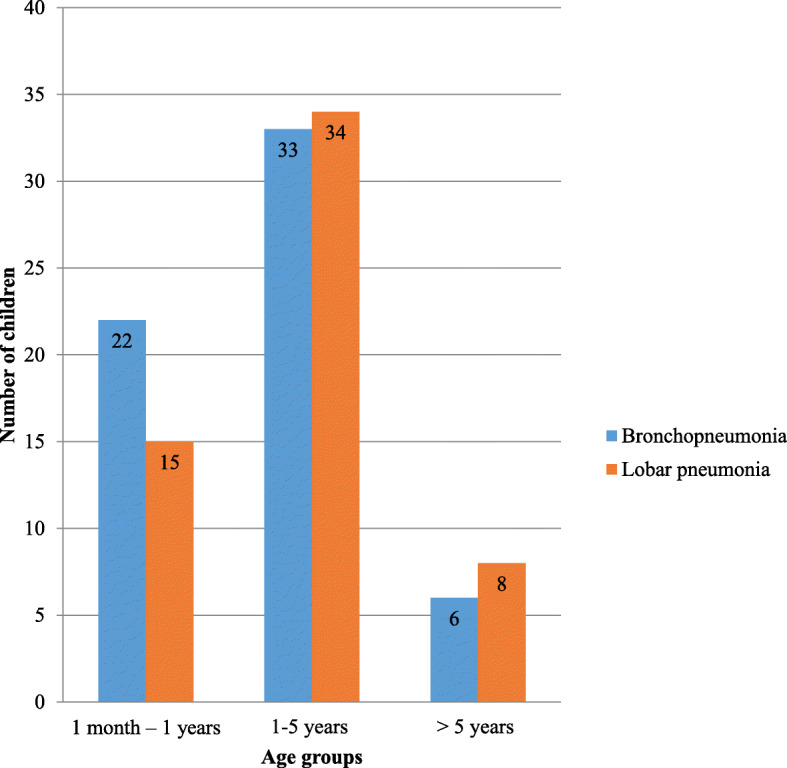
Number of children with bronchopneumonia and lobar pneumonia

In the study population, 48% were diagnosed as having bronchopneumonia and 45% as lobar pneumonia. 7% had atypical pneumonia. Majority of the patients with both bronchopneumonia and lobar pneumonia were in the pre-school age group (Fig. 1).

**Fig. 2:**
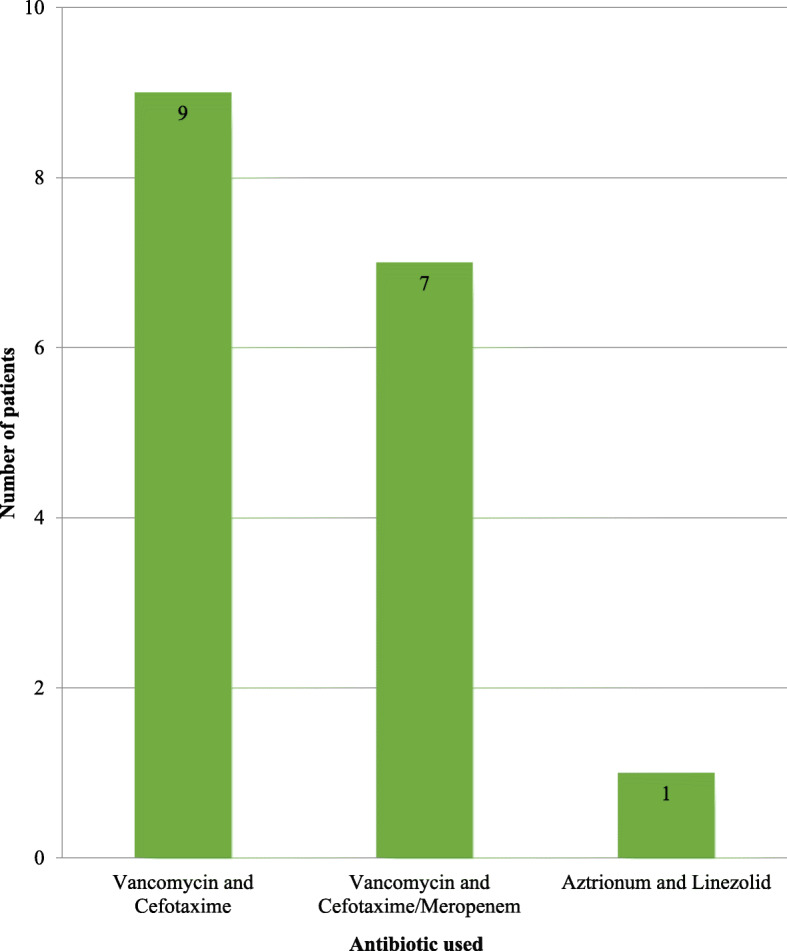
Number of patients treated with different antibiotic combinations beyond first-line antibiotics

According to the received drug, 35% (44) of the patients had only first-line intravenous antibiotics and made a complete recovery. 65% (83) of patients were prescribed drugs other than cefuroxime, cefotaxime, ceftriaxone or co-amoxiclav according to the unit policy due to inadequate response or complications. Nine of those patients were treated with Vancomycin and Cefotaxime. Seven patients were treated with Vancomycin and Cefotaxime/Meropenem. Aztreonam and Linezolid combination was given to one patient (Fig. 2).

We have analyzed the type of pneumonia, white cell count, C reactive protein levels and radiological findings in the two groups of patients- who recovered with first line of antibiotics and those who needed beyond. There was no statistical difference in the two groups for any one of the parameters (Table 1).

On discharge, 36% (46/127) of the patients were given oral antibiotics to complete the course of treatment.

When considering the treatment settings, 58% (73) of the patients were treated in the normal acute ward setting while 40% (51) of the patients were treated in the High Dependency Unit (HDU) with supplemental oxygen and close monitoring. 2% (3) needed admission to the Intensive Care Unit (ICU) according to the unit policy which included multi-organ dysfunction and difficulty in maintaining oxygenation. One of them needed invasive ventilation. The others were treated under less intensive care settings in the acute ward setting.

Out of those who received oxygen, 59% (30/51) were given oxygen therapy for 1–3 days. 15 (29%) were given for 4–6 days. Only 4 (8%) children have received oxygen for more than 6 days. Most of the patients (19; 37%) needed the highest amount of oxygen (O_2_) therapy during the 5th-9^th^day of illness. Maximum O_2_ rate that was received by most of the patients was 2 L/min via face mask (Table 2).

**Table 1:** Features of the patients who received first line antibiotics and those who received antibiotics beyond first line

	Received 1st line antibiotics(*n* = 44)	Received 2nd line antibiotics or beyond(*n* = 83)	
White cell count			
Normal	65.9% (29)	53.02%(44)	*P* = 0.2
Abnormal	29.55%(13)	31.32% (26)	
Not available	4.55% (2)	15.66%(13)	
Mean CRP level	53.86 mg/l	54.55 mg/l	*P* = 0.57
CRP > 25	50% (21)	44.28% (31)	*P* = 0.81
Type of pneumonia			
Bronchopneumonia	52.27%(23)	45.79%(38)	*P* = 0.9
Lobar pneumonia	47.73%(21)	43.37%(36)	P = 0.9
Atypical Pneumonia		10.84% (9)

**Table 2:** Characteristics of study population treated in HDU (*N* = 51)

Characteristics	Category	Count	Percentage
Duration of oxygen therapy in HDU (days)	< 1	2	3.92%
	1–3	30	58.82%
	4–6	15	29.41%
	> 6	4	7.84%
Day of the illness when the maximum O_2_ therapy was needed	1–4	13	25.49%
	5–9	19	37.25%
	10–15	8	15.69%
	> 15	3	5.88%
	N/A	8	15.69%
Maximum O_2_ rate (L/min)	1	14	27.45%
	2	19	37.25%
	3	7	13.73%
	4	3	5.88%
	N/A	8	15.69%

**Fig. 3:**
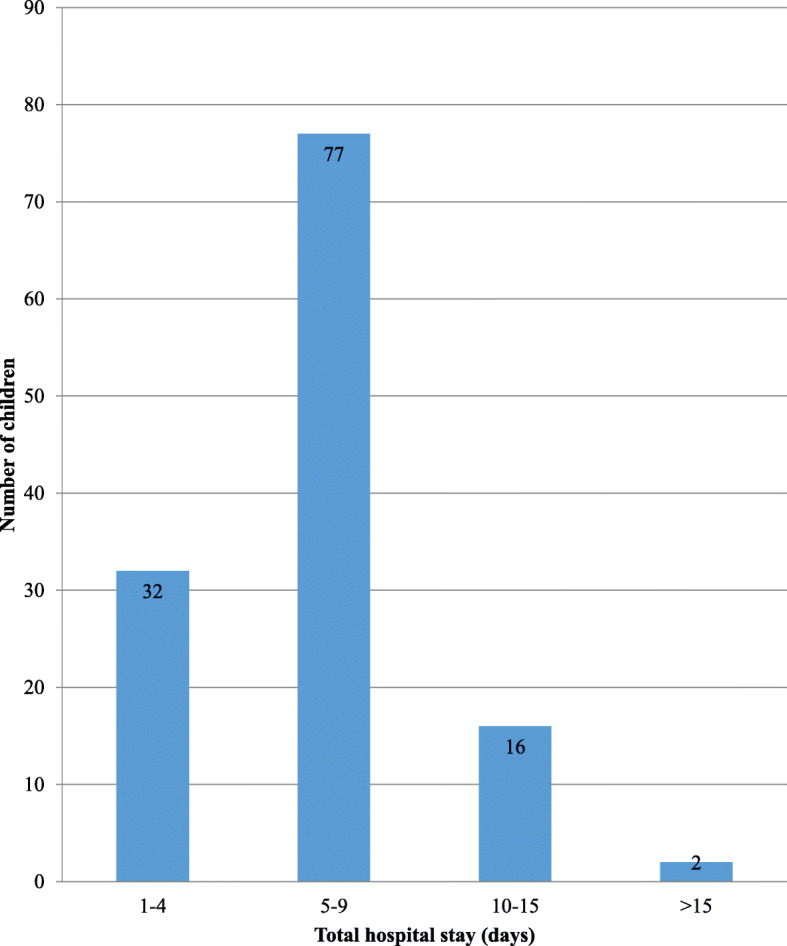
Number of children and total hospital stay

The majority of the patients (77; 61%) needed hospital stay for more than 5 days. Nearly a quarter of the total number of patients (32) who were admitted was discharged within the first 4 days of the admission (Fig. 3).

## Discussion

Pneumonia is a disease with high morbidity and mortality and continues to be an important disease adding to the disease burden of children. Sri Lankan context is not different. Measures including the introduction of pneumococcal vaccine have helped reduce the disease burden in developed countries but it continues to be a very important public health problem in those countries. Pneumonia kills 2400 children of less than 5 years of age each day [17]. Rudan et al estimated the median incidence of community-acquired pneumonia for developing countries was 0.28 episodes per child-year [18]. The value is almost similar to the estimates of the Child Health Epidemiology Reference Group (CHERG) in 2001 established by WHO [19], where the incidence of clinical pneumonia in children aged less than 5 years in developing countries worldwide, is close to 0.29 episodes per child-year. This equates to 151.8 million new cases every year, 13.1 million or 8.7% of which are severe enough to require hospitalization [18].

Sri Lanka has a Gross Domestic Product (GDP) per capita income of US $ 3926.20 in the year 2017 and the country spends 3.5% of GDP on health. Successive governments have maintained the budgetary allocations so that the country has advanced in the provision of health care to its people [20].

This study attempted to fill deficient knowledge gaps in our status of childhood community acquired pneumonia. According to the results the disease contributes to a noteworthy number of admissions among the other acute admissions.

In a study in Germany, 54% of those getting admitted with community acquired pneumonia were having bronchopneumonia. In our study, the value is 48% [21]. About 2/3 of the patients admitted with community-acquired pneumonia needed second-line of intravenous antibiotics suggesting the possibility of resistance to cephalosporins and co-amoxiclav. Pneumococcal infections caused 60,000 cases of invasive disease each year in the United States until 2000.Up to 40% of these infections were caused by pneumococcal bacteria that were resistant to at least one antibiotic. The numbers dropped fast following the introduction of pneumococcal vaccines [22].

The country does not have a well-implemented antibiotic policy and a GP system thus there is widespread overuse of antibiotics. The patients do not generally carry information on antibiotics they have been dispensed with. The results indicate the need for a proper antibiotic policy and a GP system for the country. Mortality from pneumonia is very minimal in developed countries. The estimate of mortality due to the illness has been predicted by several researchers [23]. A multiple-cause model that analyzed 38 more recent studies (average mid study surveillance year of 1990) from sub-Saharan Africa and South Asia, in countries with mortality rates for children aged less than 5 years of at least 26 per 1000 live births, predicted a similar number of deaths attributable to pneumonia (i.e. approximately 1.8 million under-5 pneumonia deaths in these two regions in the year 2000) [24].

Though there was no mortality recorded in the current study, there is significant morbidity and a noteworthy burden on the personal and national economy. In our study, 61% of the patients needed a hospital stay for 5–10 days.

## Conclusions

Community acquired paediatric pneumonia in the given population has significant associated morbidity but not mortality. The need for the second-line of intravenous antibiotics in many patients may indicate antibiotic resistance. Introduction of a national antibiotic policy and a GP system will help the cause.

## Data Availability

Data will be available on request to the main author at sanathusara@yahoo.com.

## References

[1] Epid.gov.lk. (2019). [online] Available at: http://www.epid.gov.lk/web/images/pdf/wer/2016/vol_43_no_50-english.pdf.

[2] Bradley JS, Byington CL, Shah SS, Alverson B, Carter ER, Harrison C (2011). The management of community-acquired pneumonia in infants and children older than 3 months of age: clinical practice guidelines by the Pediatric Infectious Diseases Society and the Infectious Diseases Society of America. Clin Infect Dis.

[3] Mackenzie G (2016). The definition and classification of pneumonia. Pneumonia..

[4] Dasaraju PV, Liu C (1996). Infections of the respiratory system. Medical microbiology.

[5] del Valle-Mendoza J, Orellana-Peralta F, Marcelo-Rodríguez A, Verne E, Esquivel-Vizcarra M, Silva-Caso W (2017). High prevalence of mycoplasma pneumoniae and chlamydia pneumoniae in children with acute respiratory infections from Lima, Peru. PLoS One.

[6] Kaplan W, Wirtz V, Mantel A, Béatrice PS. Priority medicines for Europe and the world update 2013 report. Methodology. 2013, 29;2(7):99-102.

[7] Samarage S. Migration and human resources for health: from awareness to action. Geneva: ILO; 2006.

[8] Smith O. Documents.worldbank.org. 2018. http://documents.worldbank.org/curated/en/138941516179080537/pdf/Sri-Lanka-Achieving-pro-poor-universal-health-coverage-without-health-financing-reforms.pdf.

[9] Annual performance report. Colombo: Ministry of Health, Nutrition and Indigenous Medicine, Sri Lanka; 2017. http://www.health.gov.lk/moh_final/english/public/elfinder/files/publications/2018/AnuualPerf_Report2017-E.pdf.

[10] Goonasekara C, Dissanayake R, Karalliedde L, Kolambage S, Wickramaratne C (2016). Critical care medicine in Sri Lanka. ICU Management Pract.

[11] Lamabadusuriya SP (2000). Immunisation of children: a sound investment for the Millenium. Sri Lanka J Child Health.

[12] Senanayake H, Goonewardene M, Ranatunga A, Hattotuwa R, Amarasekera S, Amarasinghe I (2011). BJOG.

[13] Revised WHO classification and treatment of childhood pneumonia at health facilities. Geneva: World Health Organization; 2014. https://apps.who.int/iris/bitstream/handle/10665/137319/9789241507813_eng.pdf;jsessionid=187422030D910B4EDEB9A78366F1F2DF?sequence=1. 25535631

[14] BTS guidelines for the Management of Community Acquired Pneumonia in children. Thorax. 2017. https://www.brit-thoracic.org.uk/standards-of-care/guidelines/bts-guidelines-for-the-management-of-community-acquired-pneumonia-in-children-update-2011/. 10.1136/thorax.56.suppl_4.iv1 PMC1765992 11713364

[15] Virkki R, Juven T, Rikalainen H, Svedström E, Mertsola J, Ruuskanen O (2002). Differentiation of bacterial and viral pneumonia in children. Thorax.

[16] Harris M, Clark J, Coote N, Fletcher P, Harnden A, McKean M (2011). British Thoracic Society guidelines for the management of community acquired pneumonia in children: update 2011. Thorax.

[17] UNICEFDATA (2018). Pneumonia in children - UNICEF data.

[18] Rudan I, Tomaskovic L, Boschi-Pinto C, Campbell H (2004). Global estimate of the incidence of clinical pneumonia among children under five years of age. Bull World Health Organization :Int J Publ Health.

[19] Grant O. about | CHERG| child health epidemiology reference group. Cherg.Org. 2019. http://cherg.org/about.html.

[20] 2017 health SDG profile: Sri Lanka. New Delhi: World Health Organization; 2017. http://www.searo.who.int/entity/health_situation_trends/countryprofile_srl.pdf?ua=1.

[21] Weigl JA, Puppe W, Belke O, Neusüss J, Bagci F, Schmitt HJ (2005). Population-based incidence of severe pneumonia in children in Kiel, Germany. KlinischePädiatrie..

[22] Pneumococcal disease | drug resistance | antibiotic resistance | CDC. CDC.Gov. 2017 https://www.cdc.gov/pneumococcal/drug-resistance.html.

[23] McAllister D, Liu L, Shi T, Chu Y, Reed C, Burrows J (2019). Global, regional, and national estimates of pneumonia morbidity and mortality in children younger than 5 years between 2000 and 2015: a systematic analysis. Lancet Glob Health.

[24] Morris S, Black R, Tomaskovic L (2003). Predicting the distribution of under-five deaths by cause in countries without adequate vital registration systems. Int J Epidemiol.

